# Durvalumab Consolidation After Definitive Chemoradiotherapy in Patients with Unresectable Stage III Non-Small Cell Lung Cancer: A Real-World Cohort Analysis from the German Prospective, Observational CRISP Registry (AIO-TRK-0315)

**DOI:** 10.3390/cancers18142316

**Published:** 2026-07-18

**Authors:** Martin Stuschke, Michael Thomas, Andreas Gröschel, Martin Sebastian, Martin Reck, Petra Hoffknecht, Christian Grah, Lisa Spring, Martina Jänicke, Carolin Lennartz, Paula Ludwig, Annika Groth, Parvis Sadjadian, Petros Christopoulos, Daniel Christian Christoph, Frank Griesinger, Bernward Passlick, Wilfried E. E. Eberhardt

**Affiliations:** 1 Klinik und Poliklinik für Strahlentherapie, Universitätsklinikum Essen, 45147 Essen, Germany; 2 Department of Thoracic Oncology, Thoraxklinik, Heidelberg University Hospital and National Center for Tumor Diseases (NCT), NCT Heidelberg, a Partnership Between DKFZ and Heidelberg University Hospital, 69126 Heidelberg, Germany; michael.thomas@med.uni-heidelberg.de (M.T.); petros.christopoulos@med.uni-heidelberg.de (P.C.); 3 Translational Lung Research Center Heidelberg (TLRC-H), Member of the German Center for Lung Research (DZL), 69120 Heidelberg, Germany; 4 Ambulantes Lungenzentrum, 48249 Duelmen, Germany; praxis@lunge-duelmen.de; 5 University Hospital, Goethe University Frankfurt, Frankfurt Cancer Institute, 60590 Frankfurt am Main, Germany; sebastian@med.uni-frankfurt.de; 6 Pneumologisch-Onkologische Abteilung, Airway Research Center North (ARCN), German Center for Lung Research (DZL), LungenClinic Grosshansdorf, 22927 Grosshansdorf, Germany; m.reck@lungenclinic.de; 7 Klinik für Thoraxonkologie, Niels-Stensen-Kliniken, Franziskus-Hospital Harderberg, 49124 Georgsmarienhütte, Germany; petra.hoffknecht@niels-stensen-kliniken.de; 8 Pneumologie-Lungenkrebszentrum, Gemeinschaftskrankenhaus Havelhöhe, 14089 Berlin, Germany; christian.grah@havelhoehe.de; 9 Clinical Epidemiology and Health Economics, iOMEDICO, 79106 Freiburg im Breisgau, Germany; lisa.spring@iomedico.com (L.S.); martina.jaenicke@iomedico.com (M.J.); 10 Biostatistics, iOMEDICO, 79106 Freiburg im Breisgau, Germany; carolin.lennartz@iomedico.com; 11 AIO-Studien-gGmbH, 14057 Berlin, Germany; paula.ludwig@aio-studien-ggmbh.de (P.L.); annika.groth@aio-studien-ggmbh.de (A.G.); 12 Universitätsklinik für Hämatologie, Onkologie, Hämostaseologie und Palliativmedizin, Johannes Wesling Klinikum, Universitätsklinikum der Ruhr Universität Bochum, 32429 Minden, Germany; parvis.sadjadian@muehlenkreiskliniken.de; 13 Klinik für Pneumologie & Infektiologie, Sektion Thorakale Onkologie, Thoraxzentrum Ruhrgebiet, Evangelisches Krankenhaus Herne, 44651 Herne, Germany; d.christoph@evk-herne.de; 14 Pius-Hospital Oldenburg, Universitätsklinik für Innere Medizin, University Medicine Oldenburg (UMO), 26121 Oldenburg, Germany; frank.griesinger@pius-hospital.de; 15 Medizinische Fakultät, Albert-Ludwigs-Universität Freiburg, Klinik für Thoraxchirurgie, Universitätsklinikum Freiburg, 79106 Freiburg im Breisgau, Germany; bernward.passlick@uniklinik-freiburg.de; 16 Thorakale Onkologie, Westdeutsches Lungenzentrum am Universitätsklinikum Essen, Ruhrlandklinik, 45147 Essen, Germany; wilfried.eberhardt@uni-due.de

**Keywords:** consolidation, durvalumab, NSCLC, real-world

## Abstract

Background/Objectives: Following findings of the PACIFIC trial, consolidation therapy with durvalumab was established as standard of care for patients with unresectable, stage III non-small cell lung cancer (NSCLC) and no disease progression after chemoradiotherapy (CRT). This study evaluated real-world effectiveness of durvalumab consolidation in clinical routine in Germany. Methods: Patients with unresectable, stage III NSCLC enrolled in the prospective, multicenter CRISP registry (August 2018–June 2023) were classified into two subgroups: treated with/without durvalumab consolidation therapy. Following stabilized inverse probability of treatment weighting, progression-free survival (PFS) and overall survival (OS) were assessed. Results: This analysis included 231 patients, 124 with and 107 without durvalumab consolidation therapy. In the two subgroups (with/without durvalumab), median age was 67.2/64.6 years, 51.6%/47.7% were female, and 83.1%/86.0% had Eastern Cooperative Oncology Group performance status ≤1. Most patients (87%) received concurrent CRT. In the weighted analysis, median PFS was significantly longer with durvalumab consolidation (18.6 months, 95% CI [12.8, 24.5] vs. 7.5 months, 95% CI [5.5, 8.4]; HR 0.52, 95% CI [0.37, 0.73]); median OS was numerically longer but did not reach statistical significance (34.4 months, 95% CI [25.4, not available/not reached] vs. 27.5 months, 95% CI [17.4, 59.6]; HR 0.67, 95% CI [0.44, 1.02]). Conclusions: This real-world study confirmed significant PFS improvements with durvalumab consolidation in unresectable, stage III NSCLC, with effect sizes comparable to PACIFIC. Although OS differences were not statistically significant, these findings provide valuable real-world evidence supporting the effectiveness of durvalumab consolidation in routine clinical practice.

## Simple Summary

Durvalumab consolidation therapy is the standard for patients with unresectable, stage III programmed cell death-ligand 1 expressing non-small cell lung cancer (NSCLC) without epidermal growth factor receptor mutation, who completed chemoradiotherapy without disease progression and relevant pulmonary toxicity. This study evaluated durvalumab effectiveness in German routine care. We analyzed 231 patients from the prospective CRISP registry (124 with durvalumab treatment, 107 without) using statistical weighting to balance patient characteristics. We found that durvalumab significantly extended time to disease progression (18.6 vs. 7.5 months), with effect sizes comparable to the pivotal PACIFIC trial. Patients receiving durvalumab also showed longer overall survival (34.4 vs. 27.5 months), though this difference was not statistically significant. These findings are clinically relevant as they confirm the effectiveness of durvalumab in a real-world setting across heterogeneous patient populations. This study provides supportive real-world evidence for durvalumab consolidation in patients with unresectable, stage III NSCLC.

## 1. Introduction

Lung cancer remains the most prevalent cause of cancer-related mortality worldwide, with almost 2.5 million new cases diagnosed and over 1.8 million deaths reported in 2022 [1]. The majority of cases (more than 80%) are classified as non-small cell lung cancer (NSCLC), and approximately one-third of patients are diagnosed with locally advanced disease (stage III) [2]. Stage III comprises a very heterogeneous group of patients, with variant prognosis and an increasingly complex treatment landscape [3].

For patients with unresectable stage III NSCLC, the preferred treatment option was concurrent platinum-based chemoradiotherapy (CRT), or sequential if concurrent therapy was not tolerable [4]. However, the results of PACIFIC, a randomized phase III, placebo-controlled trial, demonstrated that consolidation therapy with the programmed cell death-ligand 1 (PD-L1) immune checkpoint inhibitor (CPI) durvalumab significantly improved overall survival (OS) and progression-free survival (PFS), while exhibiting a manageable safety profile [5,6,7,8]. Consequently, durvalumab consolidation has been globally established as the standard of care for patients with unresectable stage III NSCLC, whose disease did not progress following CRT [9,10,11,12]. Following a post hoc subgroup analysis showing improved OS in patients exhibiting PD-L1 tumor proportion score (TPS) levels of ≥1%, but not in those with a PD-L1 TPS of <1%, the European Medicines Agency (EMA) restricted approval of durvalumab consolidation therapy to patients with tumors expressing PD-L1 at a threshold of TPS ≥ 1% [13,14]. In contrast, the Food and Drug Administration (FDA) granted approval irrespective of PD-L1 expression. However, for patients with epidermal growth factor receptor (*EGFR*) mutations or anaplastic lymphoma kinase rearrangements, the benefit of durvalumab consolidation has not been demonstrated; thus alternative treatment options are needed [12,15]. In December 2024, consolidation therapy with osimertinib was approved for patients with *EGFR*-mutated unresectable stage III NSCLC [16].

The efficacy of durvalumab consolidation has been supported by the international real-world PACIFIC-R study, an observational, retrospective study of patients with unresectable, stage III NSCLC who received durvalumab through an early access program (EAP) [17,18]. Furthermore, several real-world studies have subsequently investigated the effectiveness of this treatment approach, confirming its beneficial effect [19,20]. However, the population of patients with stage III NSCLC is very heterogeneous. It is therefore of particular importance to obtain data from the analysis of different, independent cohorts, as these can provide valuable insights into the use and effectiveness of consolidation therapy with durvalumab in real-world.

The present study investigates the effectiveness of durvalumab consolidation on PFS and OS in a diverse real-world cohort, the German, prospective, multicenter tumor registry CRISP NSCLC stage I–III. We present clinical characteristics and treatment details of patients in routine care in Germany and compare the outcomes of patients treated with or without durvalumab.

## 2. Patients and Methods

### 2.1. Study Design and Cohort Definition

CRISP (AIO-TRK-0315) is an open, non-interventional, prospective, multicenter tumor registry with the main objective to assess molecular biomarker testing, treatment, and outcome of patients with NSCLC or SCLC in Germany. More than 150 certified lung cancer centers, comprehensive cancer centers, hospitals and office-based oncology practices across Germany participate in CRISP. CRISP has been reviewed by the responsible ethics committee and is registered at ClinicalTrials.gov (NCT02622581); details have been published previously [21,22,23,24,25].

The CRISP satellite NSCLC stage I–III collects data on diagnostics, patient and tumor characteristics, biomarker testing, treatment planning, all antitumoral therapies, and course of disease. Inclusion criteria for this CRISP satellite are (i) age ≥ 18 years, (ii) confirmed NSCLC stage I, II, IIIA, or IIIB/C if patient is eligible for surgery and/or CRT in a curative intention (staging according to tumor, node and metastasis (TNM) classification system of malignant tumors, UICC 8th edition [26]), (iii) ability to understand and willingness to sign written informed consent and to complete patient-reported outcome assessment instruments, and (iv) signed informed consent no later than four weeks after start of anti-tumor treatment in curative intention.

Patients included in this analysis were recruited from August 2018 to February 2020 and from December 2020 until June 2023. Data cut for the present analysis was 30 June 2024.

For patients to be considered in the statistical analysis, the following variables were required to be documented: year of birth, sex, and data on antitumoral treatment. For this study, only patients with stage III NSCLC (unresectable and one primary tumor location), with no disease progression after definitive CRT, and documented PD-L1 test result, were considered. Based on the treatment received, patients were classified into two distinct subgroups: treated with versus treated without durvalumab. Patients were classified as treated with durvalumab consolidation if at least one administration of durvalumab after completion of definitive CRT was documented, irrespective of the start interval after CRT or the dosing regimen used, and no disease progression had been documented prior to durvalumab initiation. Patients were followed until the end of the project (at least three years), or until death or lost to follow-up.

### 2.2. Stabilized Inverse Probability of Treatment Weighting

Stabilized inverse probability of treatment weighting (IPTW) was applied, to adjust for possible confounding [27]. Propensity scores were calculated by logistic regression, using the following variables: age in years (mean), sex (male), TNM stage, Eastern Cooperative Oncology Group performance status (ECOG), Charlson comorbidity index (CCI), and histology type (squamous). To assess the stabilized IPTW, the mean differences (MD) for the variables employed (standardized mean difference (SMD) for the parameter age) between the two subgroups (treated with/without durvalumab) before and after IPTW were determined.

### 2.3. Time-to-Event (TTE) Analysis

All TTE analyses were conducted using the Kaplan–Meier method [28]. PFS was defined as the interval between end of CRT and first documented tumor progression/recurrence or death of any cause, whatever occurred first. Patients without such an event at data cut were censored at last contact. OS was defined as the time from end of CRT until date of death from any cause. Patients without event at data cut were censored at last contact. The Cox proportional hazard model was used to calculate hazard ratios (HRs) including the two-sided 95%–confidence interval (CI) of the weighted TTE analyses, using robust variance estimation.

To assess the robustness of the findings, two sensitivity analyses were performed: a restricted mean survival time (RMST) analysis for OS at 6, 12, and 24 months, and a six-week landmark analysis to address potential immortal time bias, including only patients alive and progression-free at the landmark, with PFS and OS calculated from that time point onward.

### 2.4. Statistical Analysis

All statistical analyses and data visualization were performed using R software, version 4.3.2 (31 October 2023) “Eye Holes” (Platform: x86_64-pc-linux-gnu (64-bit)) [29].

## 3. Results

### 3.1. Cohort Description and Patient Characteristics

**Figure 1:**
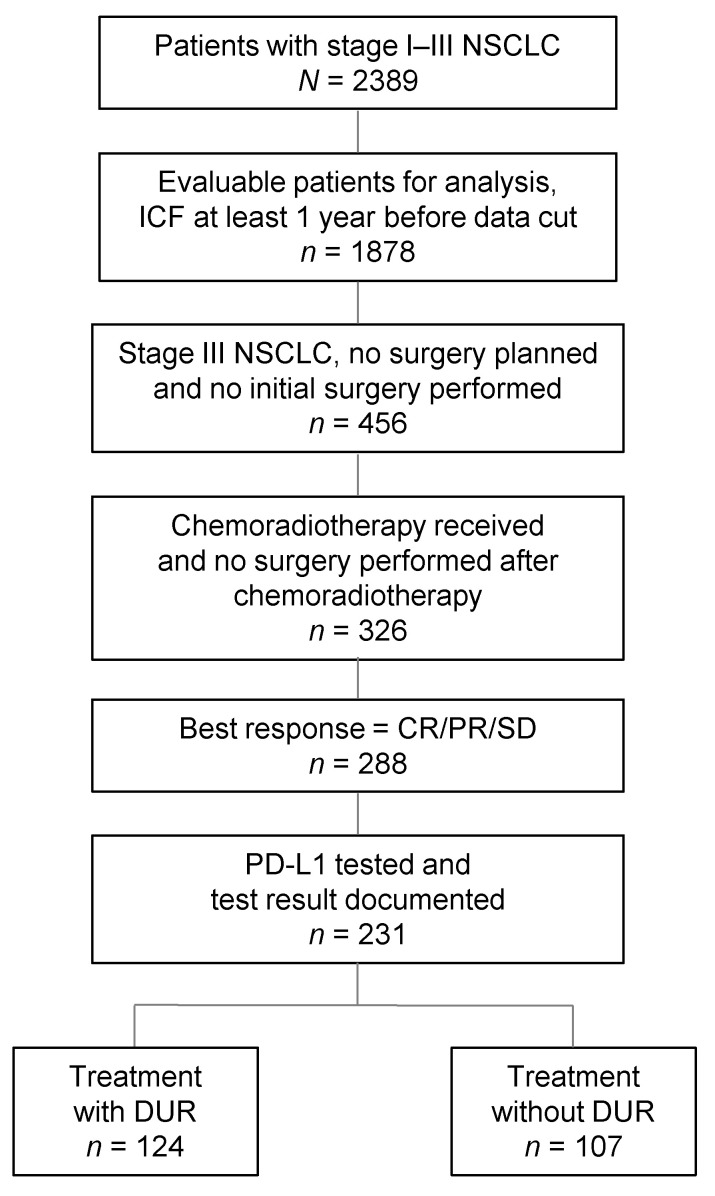
Cohort definition. Patient flow chart of all patients with early stage and locally advanced NSCLC recruited into the CRISP satellite NSCLC stage I–III from August 2018 until June 2023 (recruitment paused between February 2020 and December 2020). Patients included in this analysis: evaluable (demographics and treatment documented), stage III NSCLC, no surgical treatment planned or performed, no evidence of disease progression within definitive chemoradiotherapy, documented PD-L1 test and results. Abbreviations: CR, complete response; DUR, durvalumab; ICF, informed consent form; NSCLC, non-small cell lung cancer; PD-L1, programmed cell death-ligand 1; PR, partial response; SD, stable disease.

Between August 2018 and June 2023, 2389 patients with early stage and locally advanced NSCLC were recruited into the CRISP satellite NSCLC stage I–III by 146 sites located throughout Germany (Figure 1). A total of 1878 patients were evaluable for analysis, with a signed informed consent at least one year before data cut. The present analysis is limited to patients with NSCLC diagnosed at stage III, with unresectable tumors (no surgery planned and not initially resected), who received definitive CRT, exhibited no evidence of tumor progression following this treatment, and for whom a PD-L1 test result was documented (*n* = 231). Consolidation therapy with durvalumab was administered to 124 patients, while 107 patients were treated without durvalumab.

Patient and tumor characteristics as well as details on treatment of the total study cohort and both subgroups of patients (treated with/without durvalumab) are presented in Table 1 and Table 2, respectively. Median age at initial diagnosis was 67.2 and 64.6 years (with/without durvalumab), and 51.6%/47.7% of the patients were female, respectively. Most patients had a good to moderate ECOG performance status of ≤1: 83.1% of patients treated with durvalumab, and 86.0% of patients treated without durvalumab.

Most patients (87%) received concurrent CRT. The proportion of patients who had already received further treatment for relapsed disease was higher in the subgroup of patients treated without durvalumab (43.0% vs. 24.2%).

### 3.2. Propensity Score Weighting

**Figure 2:**
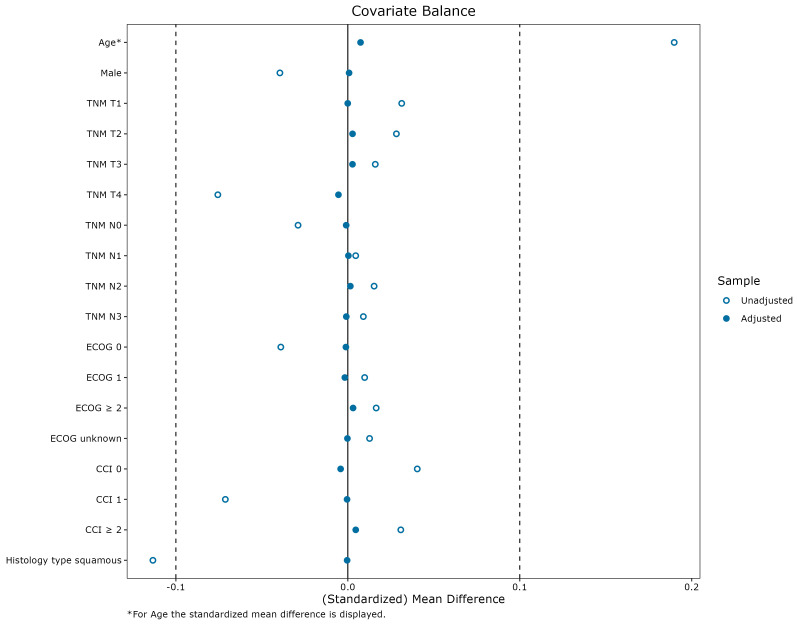
Balance of individual covariates. Love plot illustrating the MDs (SMD for the parameter age) between the two subgroups of patients analyzed (treated with/without durvalumab) before (unadjusted) and after (adjusted) propensity score weighting. Balance is defined as (S)MD < 0.1. Abbreviations: CCI, Charlson comorbidity index; ECOG, Eastern Cooperative Oncology Group; (S)MD, (standardized) mean difference; TNM, tumor, node and metastasis classification of malignant tumors according to UICC 8th edition [26].

To account for potential confounding, IPTW was used to balance baseline characteristics between the patient subgroups (with/without durvalumab). Patient characteristics before and after weighting are summarized in Supplementary Table S1. Before weighting, the largest (S)MDs were observed for age (SMD = 0.190), squamous histology (MD = −0.113), TNM T4 stage (MD = −0.076), and CCI 1 (MD = −0.071), see Figure 2. After weighting, (S)MDs were below 0.1 for all covariates included in the analysis, indicating good balance.

### 3.3. Clinical Outcome

**Figure 3:**
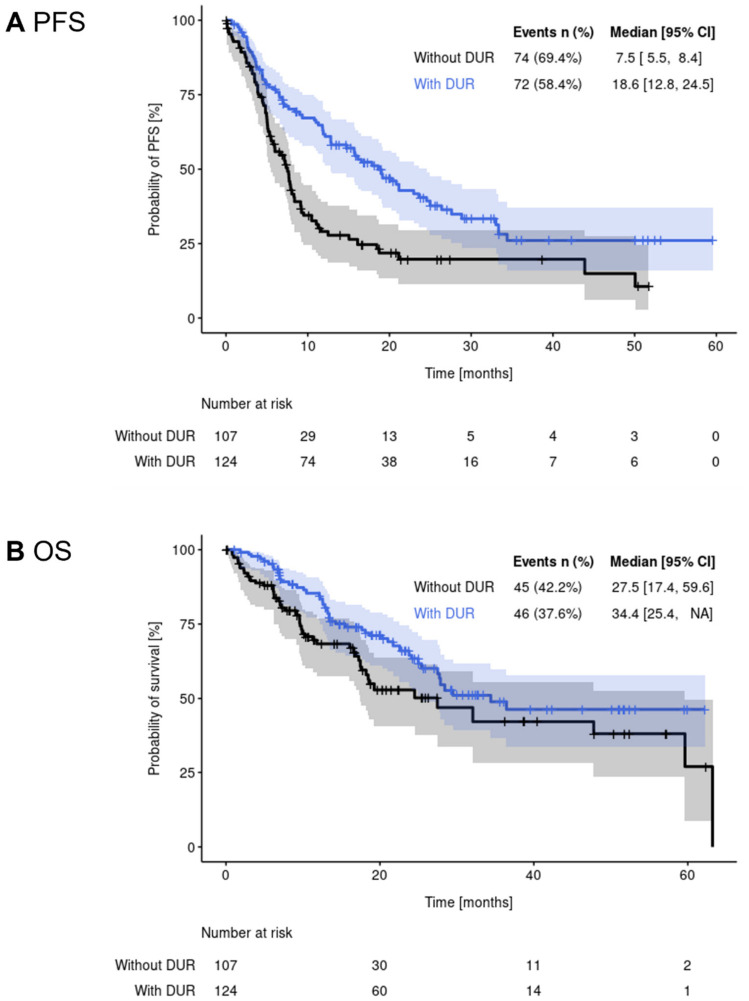
Clinical outcome of the weighted patients’ subgroups. Real-world PFS (**A**) and OS (**B**) after IPTW in patients with unresectable stage III NSCLC treated with vs. without durvalumab consolidation following CRT. Recruitment: August 2018 to February 2020, and December 2020 to June 2023. Data cut was 30 June 2024. (PFS: HR 0.52, 95% CI [0.37, 0.73]; OS: HR 0.67, 95% CI [0.44, 1.02]). Abbreviations: CI, confidence interval; DUR, durvalumab; HR, hazard ratio; NA, not available/not reached; OS, overall survival; PFS, progression-free survival.

Figure 3 presents Kaplan–Meier estimates and survival curves for real-world PFS and OS for the weighted cohort, separating patients treated with vs. without durvalumab treatment. At time of data cut, 58.4% and 69.4% of patients, respectively, experienced tumor progression or death after end of CRT. Median PFS was significantly longer for patients receiving consolidation treatment with durvalumab (18.6 months, 95% CI [12.8, 24.5] vs. 7.5 months, 95% CI [5.5, 8.4]; HR 0.52, 95%CI [0.37, 0.73], *p* < 0.001).

A total of 37.6% of patients treated with durvalumab and 42.2% of patients treated without durvalumab had died at the time of data cut. Median OS was 34.4 months (95% CI [25.4, not available/not reached (NA)]) for patients receiving durvalumab consolidation and 27.5 months (95% CI [17.4, 59.6]) without durvalumab consolidation. However, the difference between survival curves was not statistically significant by the log-rank test (*p* = 0.062) and the corresponding hazard ratio did not indicate a significant difference between the two groups (HR 0.67, 95% CI [0.44, 1.02]). To limit the influence of subsequent palliative therapies on OS interpretation, RMST was calculated at 6, 12, and 24 months as a sensitivity analysis (see Supplement Tables S2 and S3). The difference in RMST between the groups treated with vs. without durvalumab increased over time, with statistical significance reached at all three time points.

To account for potential immortal time bias related to the interval between CRT completion and initiation of durvalumab consolidation, a six-week landmark sensitivity analysis including only patients alive and progression-free at the landmark was performed; results were consistent with the main analysis, supporting the robustness of our findings.

## 4. Discussion

Based on findings from the PACIFIC trial, consolidation therapy with durvalumab has been established as the standard of care for patients with unresectable, stage III, PD-L1 positive (TPS ≥ 1%) NSCLC and no disease progression after CRT. While PACIFIC-R and other real-world studies have demonstrated favorable real-world outcomes for this treatment approach [17,18,19,20], the stage III NSCLC population is highly heterogeneous, and real-world evidence from Europe remains limited. In the present study, we evaluated the real-world effectiveness of durvalumab consolidation in clinical routine in Germany using data from a diverse cohort, enrolled in the prospective, multicenter tumor registry CRISP satellite NSCLC stage I–III. To account for potential confounding, we applied IPTW based on the most relevant prognostic factors (age, sex, tumor stage, ECOG, CCI, and histology). This approach balanced baseline characteristics between the patient subgroups (with/without durvalumab), enabling a more direct comparison of PFS and OS. Notably, the PD-L1 status was not included in IPTW as it was a selection factor for durvalumab treatment. The PD-L1 status is not a favorable prognostic factor after CRT alone and therefore cannot explain the differences in PFS found in this study [31,32,33,34].

The PACIFIC trial demonstrated significant improvements for consolidation therapy with durvalumab compared to placebo in both PFS (updated median PFS: 16.9 months, 95% CI [13.0, 23.9] vs. 5.6 months, 95% CI [4.8, 7.7]) and OS (updated median OS: 47.5 months, 95% CI [38.1, 52.9] vs. 29.1 months, 95% CI [22.1, 35.1]) [8]. Our data are largely consistent with these findings: In the weighted analysis, durvalumab consolidation significantly prolonged median PFS (18.6 months, 95% CI [12.8, 24.5]) in comparison to no consolidation (7.5 months, 95% CI [5.5, 8.4]), with an effect size (HR 0.52, 95% CI [0.37, 0.73]) similar to the PACIFIC trial (HR 0.55, 95% CI [0.45, 0.68] [8]). While median OS in our cohort was numerically longer with durvalumab consolidation (34.4 months, 95% CI [25.4, NA] vs. 27.5 months, 95% CI [17.4, 59.6]), this difference did not reach statistical significance, in contrast to PACIFIC, where median OS values were also longer than those observed in our study. However, it is widely acknowledged that patients enrolled in randomized clinical trials with strict inclusion criteria are carefully selected and thus not representative of all patients treated in clinical routine [25,35]. Whereas the PACIFIC trial enrolled only patients with ECOG ≤ 1 undergoing concurrent CRT and excluded those with grade 2 or higher pneumonitis from previous CRT [5,6], our total cohort included patients with ECOG ≥ 2 (6.5%), those receiving sequential CRT (13.0%), and patients with pneumonitis were not excluded—all factors associated with poorer survival outcomes [19,36,37].

Beyond the international real-world PACIFIC-R study, which reported a median PFS of 24.3 months (95% CI [20.3, 28.4]) and a median OS of 59.0 months (95% CI [52.7, 64.3]) with durvalumab consolidation [38], multiple real-world studies across different cohorts of patients with unresectable stage III NSCLC have demonstrated good PFS and OS values with durvalumab consolidation therapy [19,20], although with considerable variability in survival outcomes. Most of these studies focused on durvalumab-treated cohorts, with only few directly comparing outcomes with versus without consolidation therapy. In the retrospective US study SPOTLIGHT, for patients who completed concurrent CRT without progressing, median PFS was 20.0 months (95% CI [16.2, not estimable (NE)]) with durvalumab consolidation compared to 10.2 months (95% CI [6.7, 12.4]) without durvalumab, and median OS was not reached versus 24.8 months (95% CI [13.4, NE]) [39]. A German real-world study, which used propensity-score matching to compare patients with PD-L1 positive stage III NSCLC receiving durvalumab to a historical cohort treated with CRT alone, demonstrated that durvalumab consolidation significantly improved PFS and OS [40]. Real-world data from the German EAP on 126 patients receiving durvalumab consolidation showed a median PFS of 20.1 months (median OS not reached) [41], which is in line with the median PFS observed in our study, whereas, the UK real-world study CODAK observed a median PFS of 28.5 months (95% CI [16.4, not reached (NR)]) and a median OS of 35.9 months (95% CI [35.9, NR]) for patients treated with durvalumab consolidation [42]. Overall, as reviewed in [20], among real-world studies from different countries in stage III NSCLC reporting OS data (published between 2017 and 2024), median OS was 38.3 months (ranging from 18.2 to 58.7 months), which aligns with our findings. However, direct comparisons between different real-world cohorts should be interpreted with caution, as variations in baseline characteristics, treatment regimens, and follow-up duration may influence the reported clinical outcomes. In addition to ECOG and type of CRT, several prognostic factors have been identified, including age, histology, chemotherapy regimen, gender, PD-L1 expression, and tumor stage [20], which may vary considerably between cohorts.

Regarding subsequent therapies in our cohort, at time of data cut, patients treated without durvalumab consolidation more frequently received CPI-based first-line treatment for relapsed disease (31.8% vs. 11.3%). This means these patients were also exposed to immunotherapy. This difference in the timing of CPI administration may explain the convergence of OS curves over time. However, with longer follow-up, the OS advantage with durvalumab consolidation may become more evident, potentially achieving significance. This is further supported by the RMST analysis, which demonstrated statistically significant OS differences at all three time horizons, with the absolute difference increasing over time—consistent with an accumulating rather than diminishing survival benefit associated with durvalumab consolidation.

The present study has some limitations. Given the observational design of the study, there are no specifications as to the timing, frequency, or criteria of tumor assessment. Therefore, registry PFS data should be considered as the best clinical approximation and might not be directly comparable to PFS determined in clinical trials. Additionally, it is important to note that subsequent treatment may have influenced the OS outcomes observed in this study. Moreover, despite adjustment for key prognostic factors using IPTW, confounding from additional/unmeasured variables cannot be excluded. In the present analysis, only patients with documented PD-L1 test results were included. While the PD-L1 status is valuable for data interpretation, this may have affected cohort composition. However, PD-L1 testing and test results were documented for most patients in the analyzed cohort (approximately 80% of patients with no evidence of disease progression after CRT, see Figure 1). In addition, the frequency of PD-L1 testing mainly depended on the time of recruitment, with documented testing increasing over time. This suggests that substantial selection bias is unlikely. Important strengths include the prospective, longitudinal data collection from multiple sites across Germany, providing valuable real-world data from a heterogeneous patient population that reflects routine clinical practice and complements the body of evidence. IPTW adjustment minimized potential confounding and enabled direct comparison of clinical outcomes with/without durvalumab consolidation, a methodological strength rarely employed in other real-world studies, which predominantly focus on durvalumab-treated cohorts.

## 5. Conclusions

This real-world analysis from the prospective, multicenter CRISP registry demonstrates significant PFS benefits with durvalumab consolidation in patients with unresectable, stage III NSCLC, with an effect size (HR 0.52) comparable to the pivotal PACIFIC trial. While OS differences did not reach statistical significance, these findings provide important real-world evidence supporting durvalumab consolidation as an effective treatment strategy in routine clinical practice across a heterogeneous German patient population, including those with characteristics often excluded from clinical trials.

## Figures and Tables

**Table 1:** Patient and tumor characteristics.

Characteristic at Initial Diagnosis	Treatment with DUR (*n* = 124)	Treatment without DUR (*n* = 107)	Total (*n* = 231)
Age in years			
Median	67.2	64.6	66.4
25–75% quantile	62.3–73.2	59.6–70.9	61.0–71.7
<65 years	47 (37.9%)	55 (51.4%)	102 (44.2%)
≥65 years	77 (62.1%)	52 (48.6%)	129 (55.8%)
Mean (StD)	66.9 (8.03)	65.4 (8.25)	66.2 (8.15)
Sex			
Female	64 (51.6%)	51 (47.7%)	115 (49.8%)
Male	60 (48.4%)	56 (52.3%)	116 (50.2%)
Performance status			
ECOG 0	45 (36.3%)	43 (40.2%)	88 (38.1%)
ECOG 1	58 (46.8%)	49 (45.8%)	107 (46.3%)
ECOG ≥ 2	9 (7.3%)	6 (5.6%)	15 (6.5%)
Unknown	12 (9.7%)	9 (8.4%)	21 (9.1%)
Comorbidities according to CCI ^a^			
CCI 0	56 (45.2%)	44 (41.1%)	100 (43.3%)
CCI 1	41 (33.1%)	43 (40.2%)	84 (36.4%)
CCI ≥ 2	27 (21.8%)	20 (18.7%)	47 (20.3%)
Smoking status			
Current smoker	36 (29.0%)	32 (29.9%)	68 (29.4%)
Former smoker (heavy) ^b^	53 (42.7%)	54 (50.5%)	107 (46.3%)
Former smoker (intensity unknown)	11 (8.9%)	9 (8.4%)	20 (8.7%)
Former smoker (light) ^b^	12 (9.7%)	4 (3.7%)	16 (6.9%)
Never smoker	8 (6.5%)	5 (4.7%)	13 (5.6%)
Unknown	4 (3.2%)	3 (2.8%)	7 (3.0%)
Pre-therapeutic tumor stage (UICC 8th edition [26]) ^c^			
Stage IIIA	39 (31.5%)	35 (32.7%)	74 (32.0%)
Stage IIIB	62 (50.0%)	48 (44.9%)	110 (47.6%)
Stage IIIC	23 (18.5%)	24 (22.4%)	47 (20.3%)
T stage			
T1	12 (9.7%)	7 (6.5%)	19 (8.2%)
T2	29 (23.4%)	22 (20.6%)	51 (22.1%)
T3	24 (19.4%)	19 (17.8%)	43 (18.6%)
T4	59 (47.6%)	59 (55.1%)	118 (51.1%)
N stage			
N0	8 (6.5%)	10 (9.3%)	18 (7.8%)
N1	11 (8.9%)	9 (8.4%)	20 (8.7%)
N2	61 (49.2%)	51 (47.7%)	112 (48.5%)
N3	44 (35.5%)	37 (34.6%)	81 (35.1%)
Histology			
Squamous	52 (41.9%)	57 (53.3%)	109 (47.2%)
Non-squamous	72 (58.1%)	50 (46.7%)	122 (52.8%)
PD-L1 expression			
TPS ≥ 1% ^d^	117 (94.4%)	46 (43.0%)	163 (70.6%)
TPS < 1%	7 (5.6%)	61 (57.0%)	68 (29.4%)

Data are number (%), unless otherwise indicated. Abbreviations: CCI, Charlson comorbidity index; CT, computed tomography; DUR, durvalumab; ECOG, Eastern Cooperative Oncology Group; N, node; PET, positron emission tomography; PD-L1, programmed cell death-ligand 1; StD, standard deviation; T, tumor; TPS, tumor proportion score. ^a^ according to Quan et al. 2011 [30]; ^b^ former smoker (heavy) quit smoking less than 15 years ago or quit smoking but had smoked more than 10 pack years; former smoker (light) quit smoking more than 15 years before diagnosis or quit smoking and had smoked less than 10 pack years; ^c^ PET or PET/CT were documented for 76.6% of patients in both subgroups (treatment with/without DUR) ^d^ this group includes patients for whom TPS was unknown, but PD-L1 test result was documented as “positive”.

**Table 2:** Details on treatment.

Treatment Characteristic	Treatment with DUR (*n* = 124)	Treatment without DUR (*n* = 107)	Total (*n* = 231)
Details on CRT			
Type of CRT			
Concurrent CRT	51 (41.1%)	45 (42.1%)	96 (41.6%)
Induction CT + concurrent CRT	57 (46.0%)	48 (44.9%)	105 (45.5%)
Sequential CRT	16 (12.9%)	14 (13.1%)	30 (13.0%)
Total RT dose			
>50 Gy	95 (76.6%)	80 (74.8%)	175 (75.8%)
≤50 Gy	21 (16.9%)	14 (13.1%)	35 (15.2%)
Missing	8 (6.5%)	13 (12.1%)	21 (9.1%)
Type of chemotherapy within CRT			
CIS + X	57 (46.0%)	53 (49.5%)	110 (47.6%)
CIS/CAR + X ^a^	7 (5.6%)	6 (5.6%)	13 (5.6%)
CAR + X	60 (48.4%)	47 (43.9%)	107 (46.3%)
Non-platinum	0 (0.0%)	1 (0.9%)	1 (0.4%)
Details on durvalumab treatment			
Duration of discontinued treatments in days, median (25–75% quantile), (*n* = 109) ^b^	267.0 (85.0–360.0)	n/a	n/a
Time between CRT and consolidation therapy in days, median (25–75% quantile)	30.0 (15.8–50.5)	n/a	n/a
Dose intensity [mg/kg per week] ^c^ median (25–75% quantile), (*n* = 104)	5 (4–5)	n/a	n/a
Patients with early progression ^d^			
Yes	2 (1.6%)	8 (7.5%)	10 (4.3%)
No	122 (98.4%)	99 (92.5%)	221 (95.7%)
Further systemic treatment for relapsed disease			
Further systemic treatment received			
Yes	30 (24.2%)	46 (43.0%)	76 (32.9%)
CT only	13 (10.5%)	10 (9.3%)	23 (10.0%)
CPI-mono ^e^	4 (3.2%)	17 (15.9%)	21 (9.1%)
CPI + CT ^f^	10 (8.1%)	17 (15.9%)	27 (11.7%)
Target inhibitor (TI) ^g^	3 (2.4%)	2 (1.9%)	5 (2.2%)
No, FU-phase ^h^	58 (46.8%)	21 (19.6%)	79 (34.2%)
No, LTFU ^i^	9 (7.3%)	19 (17.8%)	28 (12.1%)
No, death	27 (21.8%)	21 (19.6%)	48 (20.8%)
Missing ^j^	0 (0.0%)	0 (0.0%)	0 (0.0%)

Data are number (%), unless otherwise indicated. Abbreviations: CAR, carboplatin; CIS, cisplatin; CPI, checkpoint inhibitor; CT, chemotherapy; DUR, durvalumab; FU, follow-up; Gy, gray; LTFU, lost to follow-up; n/a, not applicable; RT, radiotherapy; CRT, chemoradiotherapy; TI, Target inhibitor; X, substances other than platin. ^a^ change from CIS + X to CAR + X or vice versa within one course of treatment; ^b^ treatment interruptions were considered as ongoing treatment, treatment discontinuation was counted as event for calculation of treatment duration; ^c^ dose modifications were rarely documented (approximately 11%); ^d^ progression or death within 6 weeks of CRT end; ^e^ CPI-mono with pembrolizumab, nivolumab, atezolizumab, ipilimumab, or durvalumab individually or in combination; ^f^ excluding combinations with Tis; ^g^ TIs: capmatinib, osimertinib, tepotinib, sotorasib; ^h^ FU-phase: patients still receiving treatment for early stage cancer or in treatment-free interval after curative treatment; ^i^ patients without documented further systemic therapies whose documentation was ended because they were LTFU; ^j^ missing: patients whose documentation has ended, but no details on further systemic treatments (received or not) had been documented at the time of data cut.

## Data Availability

The patient-level data supporting the findings of the study are not openly available due to data privacy protection regulations. Enquiries regarding the data used for this study can be directed to the corresponding author.

## Supplementary Materials

The following supporting information can be downloaded at: https://www.mdpi.com/article/10.3390/cancers18142316/s1, Table S1: Balance of covariates before and after IPTW; Table S2: Restricted mean survival time analysis of overall survival after IPTW; Table S3: Difference between restricted mean survival estimates.
