# Supplementary material for: Durvalumab Consolidation After Definitive Chemoradiotherapy in Patients with Unresectable Stage III Non-Small Cell Lung Cancer: A Real-World Cohort Analysis from the German Prospective, Observational CRISP Registry (AIO-TRK-0315)
Source: Cancers (Basel). 2026 Jul 18;18(14):2316. doi: 10.3390/cancers18142316 (PMC13407345; doi:10.3390/cancers18142316)
Supplement: Supplementary file 1 [file cancers-18-02316-s001.zip › cancers-4362132-supplementary.pdf]

Supplementary Material

Supplementary Table S1. Balance of covariates before and after IPTW

	Unadjusted			Adjusted		
**Characteristic**	**Treatment with DUR**	**Treatment without DUR**	**Difference**	**Treatment with DUR**	**Treatment without DUR**	**Difference**
Age in years (mean) [a]	66.9	65.4	0.2	66.3	66.3	0
Male	48.4%	52.3%	-3.9%	50.2%	50.1%	0.1%
TNM T1	9.7%	6.5%	3.1%	8.3%	8.3%	0%
TNM T2	23.4%	20.6%	2.8%	22.2%	21.9%	0.3%
TNM T3	19.4%	17.8%	1.6%	18.1%	17.8%	0.3%
TNM T4	47.6%	55.1%	-7.6%	51.5%	52%	-0.5%
TNM N0	6.5%	9.3%	-2.9%	7.8%	7.9%	-0.1%
TNM N1	8.9%	8.4%	0.5%	8.8%	8.7%	0%
TNM N2	49.2%	47.7%	1.5%	48.6%	48.5%	0.1%
TNM N3	35.5%	34.6%	0.9%	34.8%	34.9%	-0.1%
ECOG 0	36.3%	40.2%	-3.9%	37.8%	37.9%	-0.1%
ECOG 1	46.8%	45.8%	1%	46.9%	47.1%	-0.2%
ECOG ≥2	7.3%	5.6%	1.7%	6.3%	6%	0.3%
ECOG unknown	9.7%	8.4%	1.3%	9%	9%	0%
CCI 0	45.2%	41.1%	4%	45%	45.4%	-0.4%
CCI 1	33.1%	40.2%	-7.1%	35.1%	35.1%	0%
CCI ≥2	21.8%	18.7%	3.1%	20%	19.5%	0.5%
Histology type squamous	41.9%	53.3%	-11.3%	46.8%	46.8%	0%

Abbreviations: CCI, Charlson comorbidity index; DUR, durvalumab; ECOG, Eastern Cooperative Oncology Group; TNM, tumor, node and metastasis classification of malignant tumors according to UICC 8th edition [26]

[a] for the variable age the standardized mean difference is displayed

Supplementary Table S2. Restricted mean survival time analysis of overall survival after IPTW

	**Time (months)**	**RMST (months)**
With DUR	6	5.8973
Without DUR	6	5.5461
With DUR	12	11.1964
Without DUR	12	10.1581
With DUR	24	19.9171
Without DUR	24	17.3865

Abbreviations: DUR, durvalumab; RMST; restricted mean survival time

Supplementary Table S3. Difference between restricted mean survival estimates

	**Time (months)**	**Estimate [95% CI]**	***p*-value**
RMST Dif (without vs with DUR)	6	-0.3512 **[-0.6265, -0.0759]**	0.012394
RMST Dif (without vs with DUR)	12	-1.0383 **[-1.8210, -0.2556]**	0.009321
RMST Dif (without vs with DUR)	24	-2.5306 **[-4.6673, -0.3939]**	0.020274

Abbreviations: CI, confidence interval; DUR, durvalumab; RMST Dif, difference between restricted mean survival estimates with Kaplan–Meier;
